# Dirac directional emission in anisotropic zero refractive index photonic crystals

**DOI:** 10.1038/srep13085

**Published:** 2015-08-14

**Authors:** Xin-Tao He, Yao-Nan Zhong, You Zhou, Zhi-Chao Zhong, Jian-Wen Dong

**Affiliations:** 1 State Key Laboratory of Optoelectronic Materials and Technologies & School of Physics and Engineering, Sun Yat-sen University, Guangzhou, 510275, China

## Abstract

A certain class of photonic crystals with conical dispersion is known to behave as isotropic zero-refractive-index medium. However, the discrete building blocks in such photonic crystals are limited to construct multidirectional devices, even for high-symmetric photonic crystals. Here, we show multidirectional emission from low-symmetric photonic crystals with semi-Dirac dispersion at the zone center. We demonstrate that such low-symmetric photonic crystal can be considered as an effective anisotropic zero-refractive-index medium, as long as there is only one propagation mode near Dirac frequency. Four kinds of Dirac multidirectional emitters are achieved with the channel numbers of five, seven, eleven, and thirteen, respectively. Spatial power combination for such kind of Dirac directional emitter is also verified even when multiple sources are randomly placed in the anisotropic zero-refractive-index photonic crystal.

Photonic crystals and metamaterials are artificial materials possessing fantastic properties to manipulate wave propagation beyond nature material. Recently, the concept of topological photonics gives a new way to reconsider band engineering in photonic crystals^1^,^2^,^3^,^4^,^5^,^6^,^7^,^8^. The emergence of conical dispersion at zone boundary plays an important role in the realization of photonic topological characteristics and relates to non-zero Berry phase. With opening a nontrivial gap, robust transport can be implemented in such topological systems, and it has been distinctly observed pseudospin-filtered states in microwave metacrystal^9^,^10^. Similarly, Weyl points and nodal points are theoretically predicted in time-reversal-invariant 3D and 2D photonic crystals^11^,^12^,^13^. Another kind of conical dispersion is found at the zone center, which is induced by triply accidental degeneracy in a class of high-symmetric dielectric photonic crystals^14^,^15^,^16^,^17^,^18^,^19^,^20^ and aperiodic quasi-crystals^21^. It arises to behave as a material with zero-refractive-index properties at Dirac frequency^22^,^23^,^24^,^25^,^26^. We focus on the use of conical-dispersion-induced zero-refractive-index medium, which can relieve the unwanted interference effect in directional radiation pattern when there is amount of gain medium in the directional emitter. In this way, the devices will be more efficient to be utilized for photonic integrated circuits.

In principle, homogeneous zero-refractive-index medium could be employed to design the directional emitter with flexible emission beam number and arbitrary output boundary^27^,^28^,^29^,^30^. However, as the building block of zero-refractive-index photonic crystal is discrete, the beam number and the boundary should be limited by the symmetry of photonic crystal. Isotropic conical dispersions have been found in photonic crystals with C_4v_ and C_6v_ symmetry^14^,^18^,^19^, and thus isotropic zero-refractive-index materials can be retrieved as long as the Dirac frequency is low enough to meet effective medium criterion^31^. Consequently, directional emitters of three, four, and six beams are straightforward to be achieved by using isotropic zero-refractive-index photonic crystals. But the multiport directional emitters with much larger number beams are more challenging due to symmetry broken.

In this paper, we proposed a kind of low-symmetric photonic crystals with conical dispersion only along the C_2v_ symmetry axis. This semi-Dirac point, along with single-mode character near the Dirac frequency, ensures the existence of anisotropic zero-refractive index. Two sets of semi-Dirac points are found in rhombic photonic crystals when the radii of the rods are fixed at two slightly different values. Several kinds of multidirectional emitters are demonstrated when the included angles of rhombic lattices are 72°, 51.4°, 32.7°, and 26.7°, respectively. Spatial power combination for multidirectional emission can be also observed even when multiple sources are in random positions of the anisotropic zero-refractive-index photonic crystal.

## Results

### Semi-Dirac point in rhombic photonic crystals

**Figure 1:**
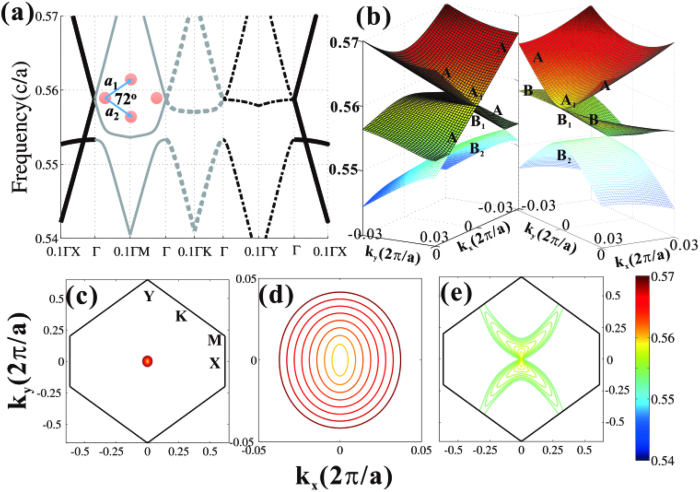
Semi-Dirac point in rhombic photonic crystal. (**a**) Two dimensional band structure along four different directions: ΓX (*k*_*y *_= 0, black solid), ΓM (*k*_*y*_/*k*_*x *_= tan18^o^, gray solid), ΓK (*k*_*y*_/*k*_*x *_= tan54^o^, gray dash), and ΓY (*k*_*x *_= 0, black dash). Inset is the lattice diagram. (**b**) Semi-X Dirac cone near Γ point. The irreducible representations of eigenmodes are labeled. (**c**) Iso-frequency contours of the frequency slightly above the semi-Dirac point of *f*_*D *_= 0.5588 *c/a*. (**d**) Zoom-in plot of (**c**) to show elliptical profiles and single-mode feature within a small **k** region. (**e**) Iso-frequency contours of the frequency slightly below the semi-Dirac point, showing the hyperbolic profiles and multi-mode at high-k components. The colorbar represents the variation of frequency.

Consider a two dimensional rhombic photonic crystal. The primitive lattice vectors are 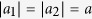 and the included angle in between is *θ *= 72°, as shown in the inset of Fig. 1(a). The radius and the permittivity of the rods are *r *= 0.195*a* and *ε = *12.5. We use plane wave expansion method to calculate the band structures^32^. Figure 1(a) shows the transverse-magnetic bulk band along four different directions. Two linear branches along the ΓX direction (*k*_*y *_= 0, black solid) intersect at Γ point at the Dirac frequency of *f*_*D *_= 0.5588*c/a*, while the dispersions of other directions (*k*_*y *_≠ 0) are quadratic. Such anisotropic feature is called semi-Dirac point^33^,^34^ and can be understood by crystallographic point group symmetry^17^. In the rhombic lattice, conical dispersion can only emerge in the directions of either ΓX or ΓY direction, as the eigenmodes at Γ point should have the same symmetries related to the non-degenerately irreducible representations of C_2v_ symmetry. Figure 1(b) highlights three bands near Γ point along the ΓX and ΓY directions in the left and right panels, respectively. At Γ point, they are **B**_**2**_**, B**_**1**_ and **A**_**1**_ modes, where **B**_**1**_ and **A**_**1**_ modes overlap by accidental degeneracy. For the ΓX direction [left panel in Fig. 1(b)], the red and yellow bands are both **A** modes as a result of conical dispersion. However, the mode symmetries along the ΓY directions are quite different, as they are **B** and **A** modes. The lack of same symmetry in the ΓY direction leads to quadratic dispersion when intersecting to each other [right panel in Fig. 1(b)]. Therefore, the conical dispersion can only be seen in the ΓX direction (*k*_*y *_= 0). Here, we define the conical dispersion along the ΓX direction as semi-X Dirac point. Another conical dispersion can emerge along the ΓY direction when the radius deceases to 0.192*a*. For the latter case, the two modes along the ΓY direction will change to both **A** modes, which is defined as semi-Y Dirac point (will discuss later).

### Anisotropic zero-refractive-index in rhombic photonic crystals

**Figure 2:**
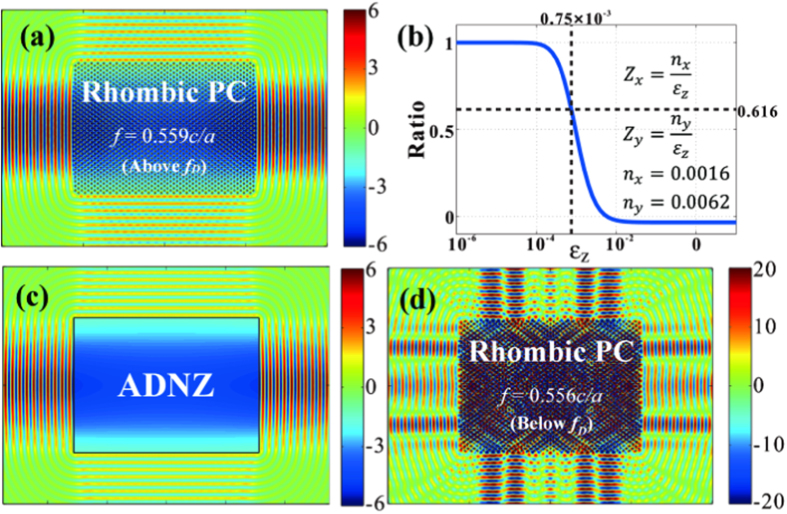
Anisotropic zero-refractive-index in rhombic photonic crystal. (**a**) Emission pattern from anisotropic zero-refractive-index photonic crystal with 72° rhombic lattice above Dirac frequency. (**b**) Output intensity ratio between the x and y directions, as a function of the permittivity of the anisotropic homogenous double near-zero (ADNZ) medium. The ADNZ material has the same effective refractive indices as (**a**) but different impedances/permittivity/permeability. (**c**) Emission pattern from the ADNZ material with the values of *Z*_*x *_= 2, *Z*_*y *_= 8, *ε*_*z *_= 0.75 × 10^−3^, *μ*_*x *_= 5.13 × 10^−2^, *μ*_*y *_= 3.25 × 10^−3^, which has the same intensity ratio as (**a**). (**d**) Emission pattern from the rhombic photonic crystal below Dirac frequency to show the absence of anisotropic near-zero and directional emission.

Conical dispersion does not necessarily indicate zero permittivity and zero permeability unless the effective medium theory can be satisfactorily applied. But it is more complicated so that the effective medium theory is not straightforward to apply to such non-square rhombic lattice. Instead, the effective refractive index may be retrieved by iso-frequency contours (IFCs) in single-mode region. Figure 1(c) shows the elliptical IFCs near Γ point above the Dirac frequency. The frequency interval is from 0.5588 to 0.57*c/a*, which is also highlighted in Fig. 1(d). It is clearly shown only one propagating mode for a given frequency, implying that the rhombic photonic crystal with semi-X Dirac point can be regarded as anisotropic homogeneous medium with near-zero refractive index when the frequency is larger than that of semi-Dirac point. For example, the effective refractive indices at *f *= 0.559*c/a* are *n*_*x *_= 0.0016 and *n*_*y *_= 0.0062 according to the IFC in Fig. 1(d). Although the refractive indices in both x and y directions are close to zero, the emission behaviors are distinct from each other due to different impedances. This is verified in Fig. 2(a). Here, we fill the rectangle device with rhombic photonic crystal. The size of device is 47*a *× 34*a*. A point source with the frequency of 0.559*c/a* is positioned in the center. In order to demonstrate the anisotropy of impedance, we put a homogenous medium with the same refractive index as the rhombic photonic crystal at *f *= 0.559*c/a*, but with different effective permittivity. Here, we use a phenomenological method to retrieve the effective permittivity *ε*_z_. We define the intensity ratio to demonstrate anisotropic behaviors,


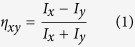


where *I*_*x*_ and *I*_*y*_ are the average exit intensity from the x and y axis, respectively. When the intensity ratios are identical between of homogeneous medium and photonic crystal, we consider that the effective permittivity of the crystal is equivalent to that of homogeneous medium. Then the other constitutive parameters can be obtained by^35^,


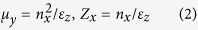



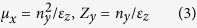


where *n, ε*, *μ*, and *Z* are the effective refractive index, permittivity, permeability and impedance, respectively. We plot the intensity ratio as the function of the effective permittivity of the homogenous medium, as depicted in Fig. 2(b). It is interesting to find that the ratio will decrease to zero as the effective permittivity changes. In particular, the ratio is the same at 0.616 between the rhombic photonic crystal [Fig. 2(a)] and the homogenous medium [Fig. 2(c)], when the homogenous medium is anisotropic double near-zero (ADNZ) material with the values of *Z*_*x *_= 2, *Z*_*y *_= 8, *ε*_*z *_= 0.75 × 10^−3^, *μ*_*x *_= 5.13 × 10^−2^ and *μ*_*y *_= 3.25 × 10^−3^. In other words, the rhombic photonic crystal with semi-X Dirac point can be viewed as the ADNZ material, and the effective impedance along the ΓX direction (with conical dispersion) is less than that along the ΓY direction (with quadratic dispersion). In addition, for the frequency interval below the Dirac frequency, the rhombic photonic crystal cannot be regarded as anisotropic zero-refractive-index material due to the multimode effects in high k components, which is also demonstrated by the hyperbolic IFCs in Fig. 1(e). Consequently, the anisotropic directional emission vanishes when the frequency is slightly below semi-Dirac point, as depicted in Fig. 2(d).

### Dirac directional emission

**Figure 3:**
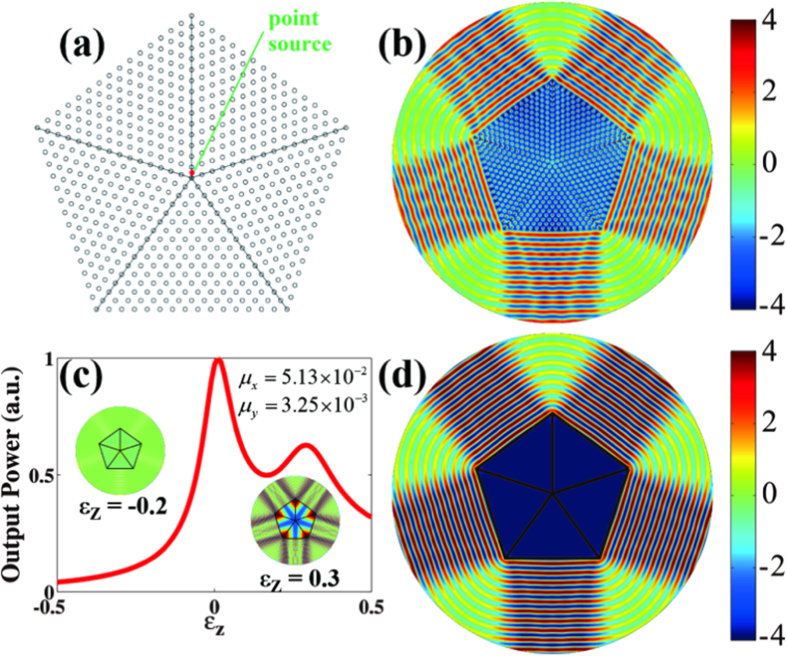
Dirac directional emission. (**a**) Schematic view of five-beam directional emitter by using anisotropic zero-refractive-index photonic crystal with 72° rhombic lattice. Five pieces of triangular-profile structure are tiled in the x-y plane to form pentagonal shape, and the exit facet is normal to the ΓX direction. (**b**) *E*_*z*_ output fields from the zero-refractive-index photonic crystal. (**c**) Output power from the pentagonal device constructed by anisotropic homogeneous mu-near-zero materials with the values of *μ*_*x *_= 5.13 × 10^−2^ and *μ*_*y *_= 3.25 × 10^−3^. The sharp peak is the result of impedance match when *ε *≈ *μ *≈ 0. (d) *E*_*z*_ fields from the pentagonal device when *ε*_*z *_= 0.75 × 10^−3^ in (c). The electromagnetic indices are taken from Fig. 2(c).

The anisotropic zero-refractive-index photonic crystals can be utilized to realize directional emission. Figure 3(a) shows the schematic view of cylinder-to-plane wave transformation for the five-beam configuration. Five pieces of isosceles triangular structures are tiled to form the pentagonal shape. The output boundary is normal to the ΓX direction. There are 16 rods from the center to the vertex of the pentagon, and hence the radius of the pentagon is *R *= 15*a*. A point source at the frequency of 0.559*c/a* (slightly above semi-Dirac point) is embedded near the center of five-beam directional emitter. The emission field pattern is shown in Fig. 3(b). Zero-phase change is observed in the structure, and the source is converted into five directional plane waves after the exit surface. To further illustrate the effective zero-refractive-index properties, we will replace the rhombic photonic crystal by five pieces of anisotropic homogeneous materials with near-zero permeability identical to Fig. 2(c). Note that the optical axes of these five pieces homogeneous materials are perpendicular to the exit facet so that the permeability tensor should make an orthogonal transformation in real space. The transformed tensor can be written as,


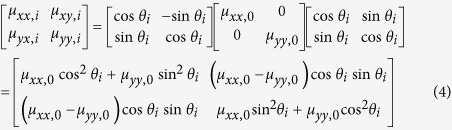


where *θ*_*i*_ = 54°, 126°, 198°, 270°, 342°, *μ*_*xx*,0 _= 5.13 × 10^−2^ and *μ*_*yy*,0 _= 3.25 × 10^−3^. Then we plot the variation of the output power as a function of the permittivity in Fig. 3(c). Because the impedance is serious mismatch to the air, the output power is close to zero when the permittivity is negative, and almost all of the energy is irradiative from the pentagon [left inset of Fig. 3(c)]. On the other hand, most energy leaks from the vertices of the pentagonal device when the permittivity is positive [right inset of Fig. 3(c)] due to anisotropy. When the permittivity approaches to zero, the output power is obviously enhanced, indicating that the impedance matches well when both ε and μ are close to 0. The corresponding field pattern of the anisotropic homogenous medium is highlighted in Fig. 3(d) at the working frequency of 0.559*c/a*, which is quite similar to the result of the rhombic photonic crystal [Fig. 3(b)].

**Figure 4:**
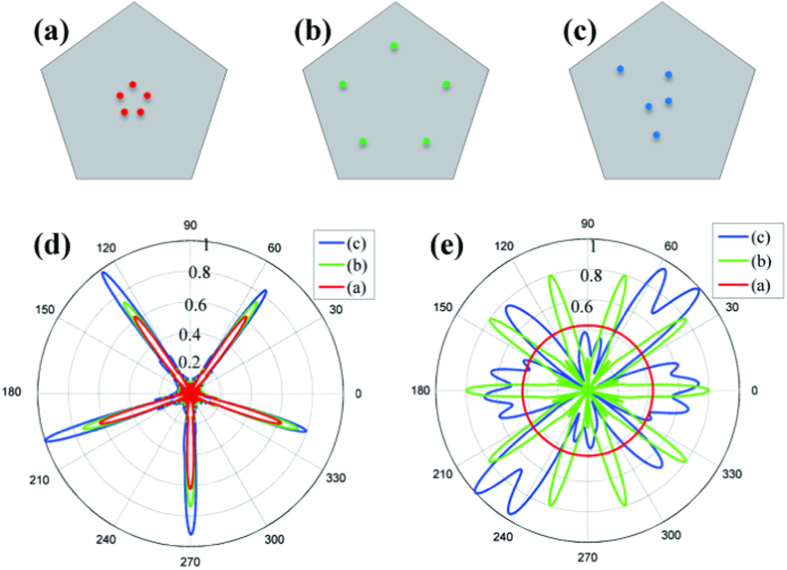
Coherent multidirectional emission in anisotropic zero-refractive-index photonic crystal with 72° rhombic lattice. (**a**)–(**c**) Locations of the five sources with same initial phases. The cases (**a**) and (**b**) have five-fold symmetry, same as the pentagonal device, while the position of the case (**c**) is random without rotational symmetry. (**d**) Far-field emission patterns. The directional patterns overlap well, regardless of all the three cases. (**e**) Same as (**d**) except in air.

In the zero-refractive-index materials, the effective wavelength of electromagnetic wave is infinite, so that spatial coherence can be well maintained inside such material. One may imagine that the directional emission would occur even when multiple sources were embedded in the anisotropic zero-refractive-index material. Figure 4 shows three kinds of source configurations in the photonic crystals. Five sources are placed symmetrically at the distance of 0.5*a* and 5.5*a* away from the pentagonal center in Fig. 4(a), while randomly in Fig. 4(c). The initial phases of the sources are identical and the working frequency is the same as Fig. 3. Normalized far-field patterns of five-beam directional emitter are plotted in Fig. 4(d), showing that the directional beams overlap well for all the three kinds of source configurations with symmetric radiation bandwidth. Note that the far-field pattern has little asymmetric intensity for the case of random configuration (blue curve) as the source locations mismatch to the symmetry of photonic crystals. In contrast, the directional feature will vanish when the sources are placed in air with improper positions. For example, omnidirectional radiation pattern is observed only when multiple sources are placed close to each other [red in Fig. 4(e)], while messy radiation patterns for other two configurations [blue and green in Fig. 4(e)].

## Discussions

**Figure 5:**
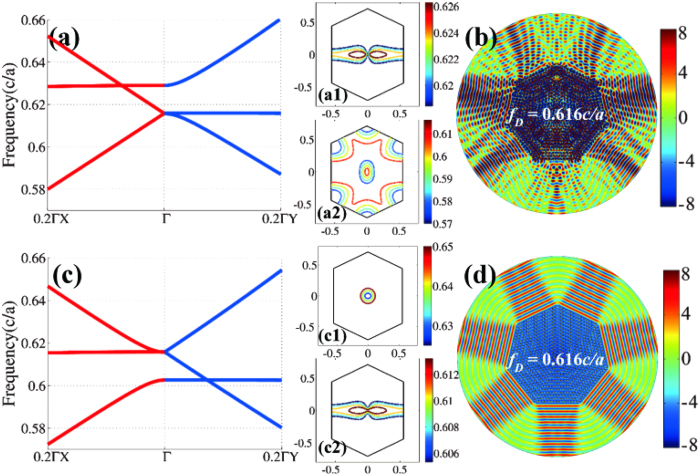
Seven-beam Dirac directional emission in photonic crystals with 51.4° rhombic lattice. (**a**) Semi-X Dirac dispersion when the radius is *r *= 0.173*a* and the permittivity is *ε *= 12.5.(**a1**)–(**a2**) The corresponding iso-frequency contours to show the multi-mode near Dirac frequency. (**b**) *E*_*z*_ fields of the heptagonal device to show the absence of zero-refractive-index feature. (**c**) Semi-Y Dirac dispersion when the radius is fixed at a slight difference value of *r *= 0.178*a*. (**c1**) Elliptical iso-frequency contours above Dirac frequency and (**c2**) Hyperbolic iso-frequency contours below Dirac frequency. (**d**) *E*_*z*_ fields of seven-beam directional emitter when zero-refractive-index is present.

The anisotropic zero-refractive-index characteristics can also be developed to directional emission with any number of beams, as long as tuning the included angle of rhombic photonic crystals. Here, we take seven-beam directional emitter for an example. The emitter is constructed by seven pieces of isosceles triangular structures. The radius of the heptagon is *R *= 15*a* and a point source is embedded near the center at the Dirac frequency. The included angle between the primitive vectors of the rhombic photonic crystal is tuned to *θ *= 51.4°. The relative permittivity is *ε *= 12.5. There are two sets of radii to ensure the presence of semi-Dirac point. When the radius is *r *= 0.173*a*, the conical dispersion exists along the ΓX direction and the semi-X Dirac point is at the Dirac frequency of 0.616*c/a* (red in Fig. 5a). However, the semi-X Dirac point does not ensure the effective zero-refractive-index properties. Single-mode character is another key factor. Figure 5(a1),(a2) depict the iso-frequency contours above and below the semi-X Dirac point, indicating that there is lack of single-mode region. Therefore, the directional emission behavior does not exist, as demonstrated in Fig. 5(b). The electric fields inside the sample are obviously distorted and the output beams are messy due to the excitation of high-k components. On the other hand, it is totally different when the radius of the rods is *r *= 0.178*a*. The conical dispersion is along the ΓY direction [Fig. 5(c)] and the elliptical iso-frequency contours appear above the semi-Dirac point, so as to guarantee the single-mode character [Fig. 5(c1),(c2)]. The anisotropic zero-refractive index is expected in this kind of photonic crystal with semi-Y Dirac point. This is verified in Fig. 5(d) that seven directional beams can be clearly observed from the heptagonal device and the phase inside the device is almost constant. Note that the single-mode character can be just found in the case of semi-Y Dirac point when the included angle is less than 60°. Thus, the directional emitter with large number of beams should be realized in the anisotropic zero-refractive-index photonic crystal with semi-Y Dirac point.

**Figure 6:**
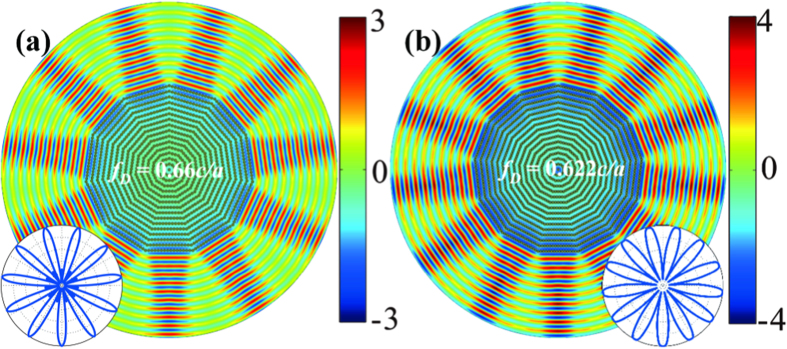
Large-beam Dirac directional emission in photonic crystals. (**a**) Eleven-beam directional emitter in photonic crystal with 32.7° rhombic lattice. The parameters are *r *= 0.186*a* and *ε *= 12.5. (**b**) Thirteen-beam directional emitter in photonic crystal with 26.7° rhombic lattice, and the parameters are *r *= 0.156*a* and *ε = *20. Blue curves in the insets are the corresponding far-field profiles in linear scale bar.

Eleven-beam directional emitter is also demonstrated in Fig. 6(a), when using the rhombic photonic crystal with the included angle of 32.7°. The radius of the rods is *r *= 0.186*a* and the permittivity is *ε = *12.5. The Dirac frequency is 0.66*c/a* and the conical dispersion is along the ΓY direction. Far-field pattern (inset of Fig. 6a) is well consistent with the near-field electric field distributions. Note that the neighboring rods may touch each other in the rhombic lattice with small included angle when the radii of rods are not so small. One of the solutions is to enlarge the permittivity of the dielectric rods, so that the semi-Y Dirac point could exist for the rods with small enough radius. Figure 6(b) shows thirteen-beam directional emission by using the rhombic photonic crystal with the included angle of 26.7°. The radius of the rods is *r *= 0.156*a*, and the permittivity is *ε = *20 The frequency of the semi-Y Dirac point is 0.622*c/a.*

**Figure 7:**
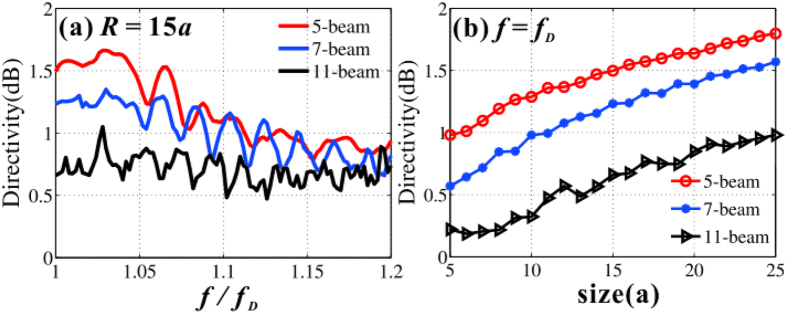
Directivity of Dirac multidirectional emitters. (**a**) Directivity of multidirectional emitters as a function of the working frequency. The polygonal radius is *R *= 15*a* for all the three emitters. (**b**) Directivity of multi-beam emitters as a function of polygonal radius *R* at the Dirac frequency.

In order to measure the directivity of emitters, a term is introduced,


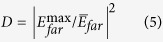


where 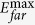 and 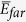 are the maximum and average values of the far field radiation patterns, respectively. Figure 7(a) shows the directivity as a function of the working frequency normalized by the Dirac frequency *f*_*D*_. It is found that the high directivity can remain in the frequency interval from *f*_*D*_ to 1.2*f*_*D*_, ensuring that the bandwidth of directional emission is substantial. This is because the directional emission can also be implemented in a small effective refractive index, not necessary for exact zero. On the other hand, one can also change the size of emitters in order to engineer the directivity, as depicted in Fig. 7(b). Optimal emitters can be retrieved by increasing the polygonal size of samples. Note that the directivity of multidirectional beam is suffered from the interferences of the adjacent beams, and longer exit boundary benefits to higher directivity. As a result, the five-beam emitter (shorter exit facet) is more directional than that of eleven beam (longer exit facet) when their sizes are same. Intuitively, when the beam number of emitters goes to infinity, multidirection turns to omnidirection with low directivity. Note also that the directivity increases with emitter size. This is because larger device size is closer to infinite crystal which is better described by effective medium theory.

In conclusion, we have studied a class of rhombic photonic crystal with C_2v_ symmetry, consisting of dielectric circular rods. The semi-Dirac point and single-mode character ensure the anisotropic zero-refractive index and the anisotropic impedances in different directions. Dirac multidirectional emitters with five, seven, eleven, and thirteen beams are achieved in such kind of low-symmetric rhombic photonic crystals with different included angles. Robustness of coherent directional emission is also found in the anisotropic zero-refractive-index photonic crystal, even when the sources are randomly placed inside the device.

## Additional Information

**How to cite this article**: He, X.-T. *et al.* Dirac directional emission in anisotropic zero refractive index photonic crystals. *Sci. Rep.* **5**, 13085; doi: 10.1038/srep13085 (2015).
